# AaMYB121, a Novel R2-MYB-like Transcription Factor, Regulates Artemisinin Biosynthesis in *Artemisia annua*

**DOI:** 10.3390/ijms26062441

**Published:** 2025-03-09

**Authors:** Dan Li, Jiaxin Song, Yueli Tang, Zeying Zhang, Chunxian Yang, Lingjiang Zeng, Xiaoqiang Liu, Min Chen, Xiaozhong Lan, Fangyuan Zhang, Zhihua Liao

**Affiliations:** 1 Integrative Science Center of Germplasm Creation in Western China (Chongqing) Science City, SWU-TAAHC Medicinal Plant Joint R&D Centre, School of Life Sciences, Southwest University, Chongqing 400715, China; lidans10@126.com (D.L.); sjxin0429@163.com (J.S.); tangyueli_999@163.com (Y.T.); zeyingz@163.com (Z.Z.); yangchunxian@163.com (C.Y.); zengling@swu.edu.cn (L.Z.); liuxq@swu.edu.cn (X.L.); 2 College of Pharmaceutical Sciences, Southwest University, Chongqing 400715, China; mmichen@swu.edu.cn; 3 TAAHC-SWU Medicinal Plant Joint R&D Centre, Key Laboratory of Tibetan Medicine Resources Conservation and Utilization of Tibet Autonomous Region, Xizang Agriculture and Animal Husbandry University, Nyingchi 860000, China; lanxiaozhong@163.com

**Keywords:** *Artemisia annua*, AaMYB121, transcription factor, artemisinin biosynthesis, promoter

## Abstract

Artemisinin, a crucial antimalarial compound synthesized in *Artemisia annua*, is tightly controlled by various transcription factors. Despite significant progress in understanding this regulatory network, further exploration of additional factors is needed to improve artemisinin biosynthesis. Here, we identified a novel R2-MYB-like transcription factor, AaMYB121, which responds to methyl jasmonate (MeJA). Overexpression of *AaMYB121* in transgenic *A. annua* plants resulted in dihydroartemisinic acid (DHAA) content being elevated 2~3 times compared to the control, while artemisinin levels increased to 1.4~2 times, significantly boosting artemisinin biosynthesis. Dual-Luciferase (Dual-LUC) assay and yeast one-hybrid (Y1H) analysis demonstrated that AaMYB121 directly binds to the promoter motifs of *DBR2* (−1146~−1103) and *ALDH1* (−1558~−1504), thereby triggering the transcriptional initiation of these genes. Notably, AaMYB121 features an elongated DNA-binding domain that specifically recognizes AT-rich *cis*-elements in the promoters of key artemisinin biosynthetic genes. These findings established AaMYB121 as a novel MYB-like transcription factor with strong potential to enhance the regulation of artemisinin production through targeted modulation, offering a valuable approach to improve artemisinin yields for therapeutic purposes.

## 1. Introduction

*Artemisia annua*, a botanically significant species deeply rooted in traditional pharmacopeia, serves as the principal botanical reservoir for the sesquiterpene lactone artemisinin [1]. This pharmaceutically vital metabolite, originally isolated through Youyou Tu’s pioneering work, has brought about a revolutionary breakthrough in the treatment of malaria, making it an indispensable therapeutic agent [2]. Despite its importance, the natural artemisinin yield in *A. annua* is relatively scarce, typically varying between 0.01% and 1% of the dry weight [3]. This limited supply has presented a significant challenge, prompting the exploration of innovative strategies to enhance artemisinin production. In recent years, various approaches have been attempted, including semi-synthetic methods using engineered yeast [4,5]. Although these methods have demonstrated partial success, their high costs have hindered their widespread application and commercial viability. Hence, improving artemisinin biosynthesis directly in the plant through advanced genetic engineering techniques remains a critical area of research.

Artemisinin biosynthesis primarily takes place in the specialized glandular secretory trichomes (GSTs) of *A. annua*, where both its production and storage occur [6]. This intricate process is governed by enzymatic cascades, including amorpha-4,11-diene synthase (ADS), cytochrome P450 monooxygenase (CYP71AV1), double bond reductase 2 (DBR2), and aldehyde dehydrogenase 1 (ALDH1) [7,8,9]. However, the expression of their corresponding genes is governed by a sophisticated network of transcription factors (TFs) that manage the entire biosynthesis process [10,11]. Among these, MYB proteins, particularly the R2R3-MYB subclass, have emerged as critical players [11]. The MYB TFs in plants typically feature 1–4 imperfect repeats, with each repeat spanning 50–53 amino acids and containing conserved tryptophan (W) residues separated by 18 to 19 amino acids (W-X_18–19_-W) [11,12]. This conserved DNA-binding domain allows MYBs to interact with specific sequences in promoter regions, thus regulating the transcription initiation of target genes [12,13]. For instance, AaMYB1 and AaMYB108 activate the expression of *ADS* and *CYP71AV1*, thereby boosting the production of artemisinin in *A. annua* [14,15]. Conversely, AaMYB15, another R2R3-MYB member with two repeats, negatively affects artemisinin synthesis by suppressing the transcription of *AaORA*, a positive regulator in the jasmonic acid (JA) signaling pathway [16]. This suppression leads to the downregulation of *ADS*, *CYP71AV1*, *DBR2*, and *ALDH1*, thereby inhibiting artemisinin generation. This regulatory complexity necessitates the discovery of novel transcriptional modulators with distinct mechanistic profiles.

In this study, we identified an unconventional R2-MYB-like TF, AaMYB121, through transcriptome clustering of GST-enriched tissues. AaMYB121 exhibits structural divergence from conventional R2R3-MYBs, suggesting alternative DNA recognition patterns. Through comprehensive molecular characterization, including promoter binding assays, gene expression studies, and metabolic profiling, we delineate its regulatory influence on artemisinin pathway components. This discovery expands the known transcriptional circuitry governing sesquiterpene biosynthesis and provides novel biotechnological targets for metabolic optimization. Furthermore, identifying AaMYB121 as a novel regulatory factor could open up new avenues for developing innovative strategies to optimize plant secondary metabolism.

## 2. Results

### 2.1. Identification of AaMYB121 as a Novel Transcriptional Factor in Artemisinin Biosynthesis

**Figure 1:**
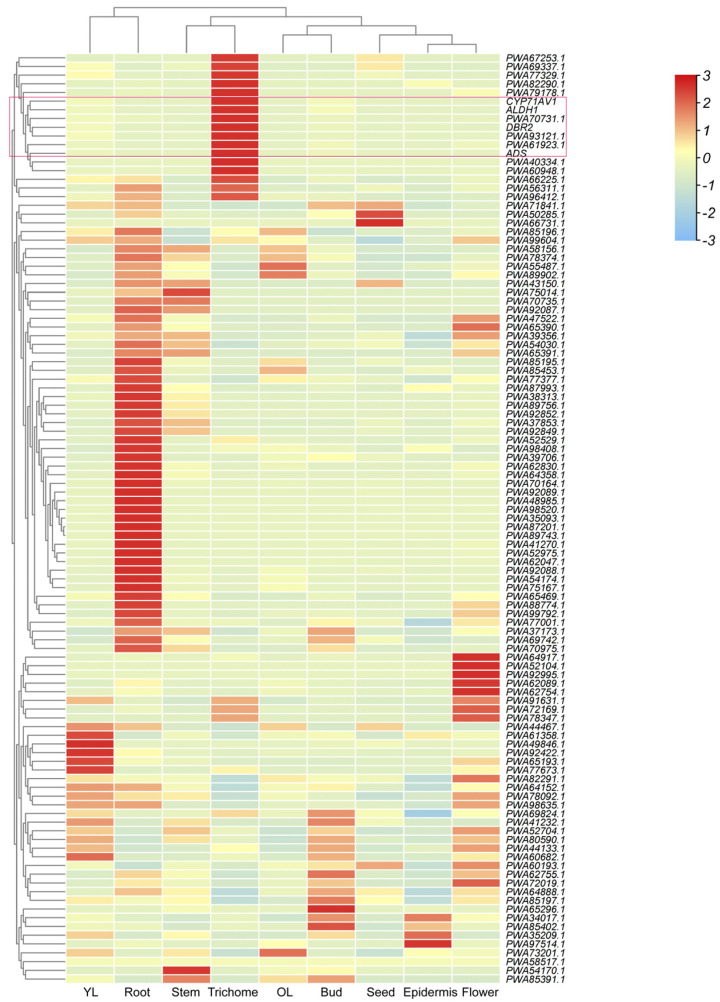
Hierarchical clustering and co-expression network analysis of *MYB*s and artemisinin pathway markers in *A. annua*. Tissue-specific expression profiles of *MYB*s and artemisinin biosynthetic genes across nine tissues: young leaf (YL), old leaf (OL), root, stem, trichome, bud, seed, epidermis, and flower. Gene identifiers were annotated on the right. The red box indicated that the *AaMYB121* (PWA93121.1) gene was clustered with core genes involved in artemisinin biosynthesis.

To uncover additional transcriptional regulators of artemisinin metabolism, we performed a clustering analysis of *MYB* family genes and artemisinin pathway markers using previously published RNA-seq data from *A. annua* in NCBI [16,17,18]. The analysis identified nine uncharacterized *MYB-like* transcripts (Gene Accession: PWA40334.1, PWA69337.1, PWA67253.1, PWA82290.1, PWA77329.1, PWA60948.1, PWA61923.1, PWA70731.1, and PWA93121.1) exhibiting strong co-expression profiles with core biosynthetic genes (*ADS*, *CYP71AV1*, *DBR2*, and *ALDH1*) (Figure 1). Strikingly, these candidates showed glandular trichome-specific expression, mirroring the spatial restriction of artemisinin biosynthesis [14]. Their spatiotemporal expression congruence with metabolic hotspots positioned these regulators as potential hubs in the artemisinin regulatory network.

**Figure 2:**
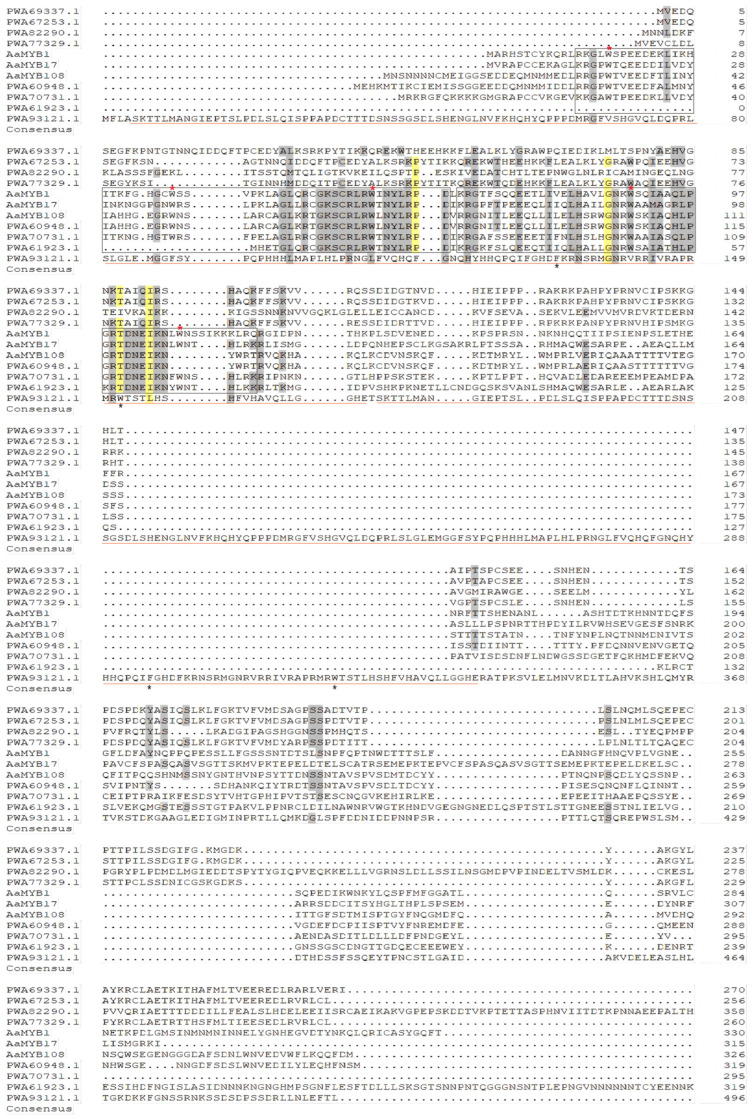
Comparisons of MYB sequences in *A. annua*. The sequences of AaMYB121 and other previously characterized MYBs in *A. annua* were analyzed. The same amino acids were shown in yellow background, while similar amino acids were shown in grey background. The conserved R2R3 motif was highlighted by a black frame. Gene identifiers for the known MYB TFs in *A. annua* were: AaMYB1 (AGR40501.1), AaMYB108 (PWA79178.1), and AaMYB17 (MW468051.1). Two repeating regions in AaMYB121 were underscored with an orange line. A red asterisk (*) indicated the tryptophan (W) residue in R2R3 motif.

Further sequence comparisons revealed marked structural conservation between PWA61923.1, PWA70731.1, and PWA60948.1 and established MYB TFs in *A. annua* (Figure 2). Notably, PWA70731.1 and PWA60948.1 display striking homology to the functionally validated AaMYB1, a canonical R2R3-MYB TF that amplifies artemisinin production through the transcriptional activation of *ADS* and *CYP71AV1* and stimulation GST development [14]. On the other hand, PWA93121.1 presents divergent domain architecture relative to standard R2R3-AaMYBs (Figure 2). Typical family members, including AaMYB1, AaMYB15, AaMYB17, and AaMYB108 maintain two conserved tryptophan-spaced motifs (W-X_18–19_-W), which are essential for DNA-binding functionality [14,15,16,19]. However, the PWA93121.1 product represents a substantially elongated polypeptide (496 residues) containing atypical MYB-related repeats that lack these diagnostic tryptophan spacing patterns. Intriguingly, two tandem repeats harbor a non-canonical F-X_20_-W motif (FKRNSRMGNRVRRIVRAPRMRW), suggesting evolutionary divergence from conventional MYB DNA-interaction modules. This divergent domain organization suggests an unconventional DNA recognition mode that might facilitate binding to atypical *cis*-elements or cooperative partnerships with non-traditional co-regulators. Given its distinctive structural features, we proposed classifying PWA93121.1 as AaMYB121, a candidate R2-MYB-like TF potentially governing artemisinin metabolism through alternative regulatory circuits.

### 2.2. Spatiotemporal Regulation of AaMYB121 Expression in A. annua

**Figure 3:**
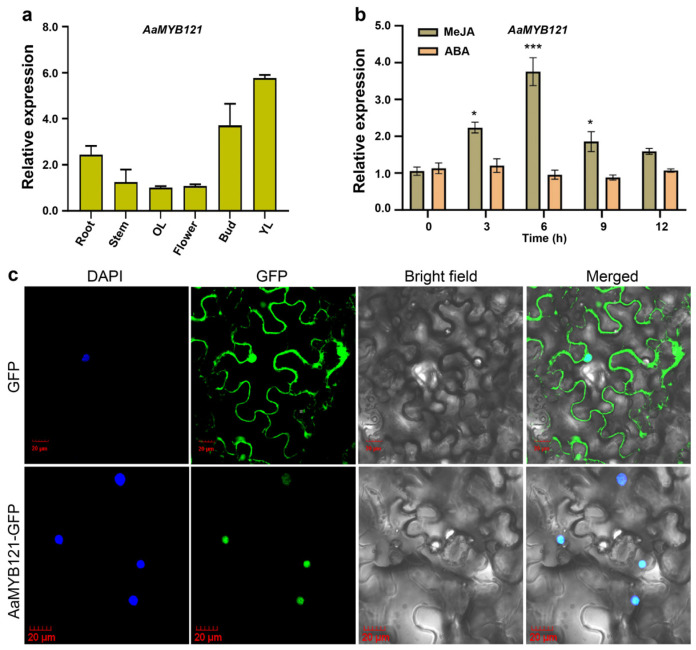
Spatiotemporal expression of *AaMYB121*. (**a**) Differential expression of *AaMYB121* across various tissues of *A. annua*. OL: old leaf; YL: young leaf. Flower was set as the control. Values: mean ± SD (n = 3). (**b**) Induction of *AaMYB121* expression in response to ABA and MeJA assessed in YL. Gene expression at the 0 h point was designated as the control. Values: mean ± SD (n = 3). * p < 0.05, *** p < 0.001, *t*-test. (**c**) Subcellular localization of AaMYB121. Transient expression of the 35S::*AaMYB121-GFP* construct was carried out in *N. benthamiana*. GFP: pHB-GFP; AaMYB121-GFP: pHB-AaMYB121-GFP. Images display DAPI, GFP, bright light, and combined fluorescence in *N. benthamiana* leaves.

Quantitative real-time PCR (qRT-PCR) was performed to delineate the spatial expression profiles of *AaMYB121* in various tissues of *A. annua*. The results revealed predominant transcript accumulation in buds and young leaves (Figure 3a), regions densely populated with GSTs [20,21]. Comparatively, other plant tissues exhibited markedly reduced expression levels. This tissue-specific expression pattern indicates that AaMYB121’s function may be associated with artemisinin production. The spatial expression correlation prompted our investigation into AaMYB121’s potential regulatory role in artemisinin biosynthesis. Given that TFs are frequently modulated by phytohormone signaling [22,23], we subsequently tested AaMYB121’s responsiveness to hormonal induction. *A. annua* plants were treated with two well-characterized phytohormones, abscisic acid (ABA) and methyl jasmonate (MeJA) [18,23], and *AaMYB121* expression in young leaves was measured. The results showed that ABA treatment did not lead to significant changes in *AaMYB121* expression (Figure 3b). However, exposure to MeJA resulted in a progressive increase in *AaMYB121* expression, peaking at 6 h post-treatment (Figure 3b). This temporal expression profile indicated that AaMYB121 is responsive to MeJA, a hormone known to regulate secondary metabolite pathways, including those related to artemisinin generation [24].

To further characterize the molecular function of AaMYB121 as a transcription factor, we constructed a plant expression vector, pHB-AaMYB121-GFP, by fusing its coding region with the *GFP* reporter gene. This vector was transiently expressed in *N. benthamiana* to determine the subcellular localization of AaMYB121 [25]. By employing DAPI (4′,6-diamino-2-phenyllindol) staining to label the nucleus specifically, we observed that, unlike the negative control GFP, which was distributed across both the nucleus and cytoplasm, the GFP signal from AaMYB121 was strictly confined to the nucleus (Figure 3c). This nuclear confinement supported the proposition of AaMYB121 functioning as a transcriptional regulator. Collectively, these findings positioned AaMYB121 as a MeJA-responsive transcriptional regulator potentially involved in modulating artemisinin biosynthesis.

### 2.3. AaMYB121 Enhances Artemisinin Biosynthesis in Transgenic A. annua Plants

**Figure 4:**
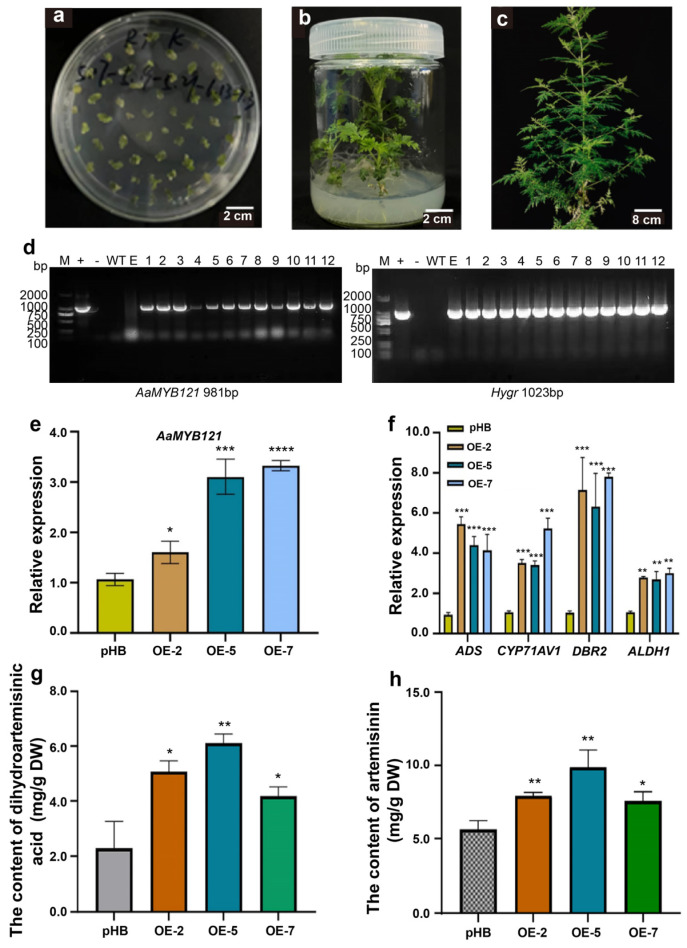
Dihydroartemisinic acid and artemisinin accumulation in transgenic *A. annua*. (**a**) Regeneration bud formation in transgenic *A. annua*. (**b**) Root development in transgenic *A. annua*. (**c**) Growth performance of transgenic *A. annua*. (**d**) Molecular confirmation of transgene integration. +: positive control; -: blank control; WT: wild-type *A. annua*; E: *A. annua* with empty vector; 1–12: transgenic lines carrying *AaMYB121*. (**e**) Expression analysis of *AaMYB121* in transgenic *A. annua*. pHB: *A. annua* plants with pHB; OE, transgenic *A. annua* overexpressing *AaMYB121*. (**f**) Expression profiles of artemisinin pathway genes in transgenic *A. annua*. (**g**) Measurement of dihydroartemisinic acid in transgenic *A. annua.* (**h**) Quantification of artemisinin in transgenic *A. annua.* All data are means ± SD (n = 3). * p < 0.05, ** p < 0.01, *** p < 0.001, **** *p* < 0.0001, *t*-test.

To assess the involvement of AaMYB121 in artemisinin biosynthesis, we utilized *Agrobacterium tumefaciens* EHA105 for genetic transformation and developed *A. annua* transgenic plants overexpressing *AaMYB121* (Figure 4a–c) [26,27]. The pHB vector incorporating the 35S promoter-driven *AaMYB121* was adopted to facilitate strong expression in *A. annua* [18]. Transgenic lines were selected by culturing explants on a hygromycin-containing medium and a total of 12 independent transgenic lines were successfully established for further analysis (Figure 4d). Next, we measured the expression levels of *AaMYB121* alongside the key genes involved in artemisinin biosynthesis in three randomly chosen transgenic lines (OE-2, OE-5, and OE-7). The qRT-PCR results demonstrated a consistent and significant upregulation of *AaMYB121* in all transgenic lines compared to control plants harboring the empty vector, confirming the successful overexpression of *AaMYB121* (Figure 4e). Notably, this genetic manipulation concurrently resulted in a simultaneous increase in the expression of several important biosynthetic genes, including *DBR2*, *ALDH1*, *CYP71AV1*, and *ADS* (Figure 4f). Among them, the most striking increase was observed for *DBR2*, which exhibited an approximately 8-fold higher expression in transgenic lines compared to control plants (Figure 4f). Therefore, the findings suggested that AaMYB121 upregulates the expression of the key genes in artemisinin biosynthesis, especially *DBR2*, thereby enhancing the generation of artemisinin.

To further test the hypothesis, we quantified the accumulation of dihydroartemisinic acid (DHAA) and artemisinin in transgenic plants utilizing HPLC analysis [27,28]. In the artemisinin biosynthetic pathway, farnesyl diphosphate (FPP) is sequentially converted into DHAA through the catalytic actions of ADS, CYP71AV1, DBR2, and ALDH1 enzymes [29]. Subsequently, DHAA is enzymatically converted to artemisinin, the final product of this pathway. The production of artemisinin is affected by various environmental factors, particularly light intensity [30,31]. Since artemisinin is the end product and its synthesis is influenced by environmental conditions, the measurement of DHAA, a direct metabolite of ALDH1, can effectively indicate fluctuations in the associated gene expression. The results revealed that DHAA levels in the leaves of transgenic plants were markedly higher, 1–2 times that of control plants, reaching approximately 6 mg/g dry weight (DW) (Figure 4g). This suggested that overexpression of *AaMYB121* promotes the conversion of FPP to DHAA, a crucial intermediate in artemisinin biosynthesis, by enhancing the expression of pathway-related enzymes. Notably, this increase in DHAA accumulation indicates that AaMYB121 may play a critical role in facilitating the early steps of the pathway, particularly in enhancing the activity of ADS, CYP71AV1, DBR2, and ALDH1. Furthermore, the determination of artemisinin content in transgenic plants revealed a significant enhancement, with artemisinin concentrations approaching 10 mg/g DW, almost doubling the yield compared to control plants (Figure 4h). This phenomenon suggested that the increased DHAA is efficiently converted into artemisinin in *A. annua*, leading to a higher overall production of the compound. The enhanced artemisinin accumulation in transgenic lines offered further proof that AaMYB121 enhances the biosynthetic pathway by stimulating the expression of critical genes responsible for converting FPP to artemisinin.

### 2.4. AaMYB121 Activates the Transcription of Artemisinin Pathway Genes

**Figure 5:**
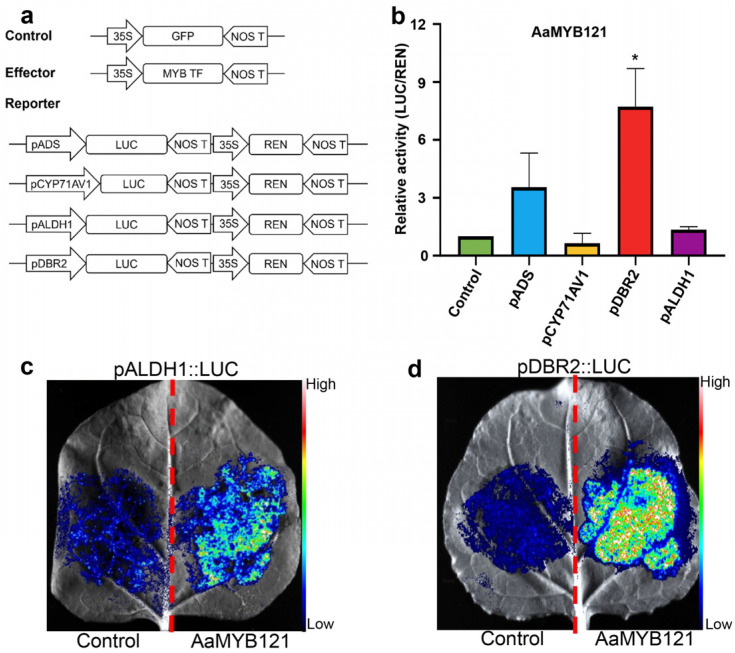
AaMYB121 activates the promoters of artemisinin biosynthetic genes. (**a**) Structural representation of effectors and reporters in Dual-LUC assays. LUC, firefly luciferase; REN, renilla luciferase; pADS, pCYP71AV1, pDBR2 and pALDH1: the promoters of the respective genes; MYB TF: AaMYB121. (**b**) Determination of promoter activity. Relative LUC activity was standardized against REN values (LUC/ REN). GFP effector served as a negative control. Values: mean ± SD (n = 5). * p < 0.05, *t*-test. (**c**,**d**) Chemiluminescence detection of *ALDH1* and *DBR2* promoter activation. The left and right sides of the red dotted line indicated the effects of the control and AaMYB121 on promoter activation, respectively.

To further clarify the regulatory impact of AaMYB121 on artemisinin synthesis, we utilized the Dual-Luciferase (Dual-LUC) assay, a widely recognized method for evaluating transcriptional activation in plant systems [16,18,23]. In this experiment, effector constructs were first generated by cloning the coding *AaMYB121* sequence into the pHB-GFP vector, controlled by the 35S promoter, to enable the production of AaMYB121 protein in plant cells (Figure 5a). As a negative control, the empty pHB-GFP vector was included to eliminate the interference of background reporter gene activity. Concurrently, reporter constructs were prepared by individually placing the promoters of four essential genes involved in artemisinin biosynthesis (*ADS*, *CYP71AV1*, *DBR2*, and *ALDH1*) upstream of the firefly luciferase (*LUC*) gene within the pGreenII0800-LUC vector (Figure 5a). These promoters contain *cis*-elements that can potentially be recognized by transcription factors, making them suitable candidates for assessing the regulatory effects of AaMYB121. The effector and reporter constructs were subsequently co-infiltrated into the leaves of *N. benthamiana*, a plant frequently employed in transient gene expression assays [16,18,25]. The results from the Dual-LUC assay revealed a significant regulatory impact of AaMYB121 on the promoters of artemisinin-related genes (Figure 5b). Notably, compared to the negative control, AaMYB121 could induce approximately an 8-fold increase in *DBR2* promoter activity. This suggested that AaMYB121 binds to specific *cis*-elements within the *DBR2* promoter, thereby recruiting the transcriptional machinery and enhancing transcriptional initiation. In addition to the robust activation of the *DBR2* promoter, AaMYB121 also seemed to exert a positive impact on the promoters of *ADS* and *ALDH1*. However, compared to *DBR2*, the activation degree for these genes was less pronounced. This indicated that AaMYB121 may affect the transcription of several genes in artemisinin biosynthetic pathway, albeit to varying extents.

To further validate these results, we carried out LUC luminescence detection. The luminescence signals from the leaves co-expressing *AaMYB121* and the reporter constructs showed that AaMYB121 only specifically enhanced the transcriptional activities of the *ALDH1* and *DBR2* promoters, with the most significant activation observed in the *DBR2* promoter (Figure 5c,d). The results indicated that the Dual-LUC assay results may have shown some bias, as AaMYB121 did not seem to activate the transcription of *ADS* as initially anticipated. Therefore, while AaMYB121 may indirectly influence the *ADS* gene, its primary effect appears to be the direct activation of *ALDH1* and *DBR2.* Collectively, these findings supported the role of AaMYB121 as a transcriptional activator modulating the expression of *ALDH1* and *DBR2*. Through these direct and possibly indirect regulatory actions, AaMYB121 likely plays a pivotal role in modulating the artemisinin biosynthetic pathway, contributing to the orchestration of artemisinin formation in *A. annua*.

### 2.5. Direct Interactions of AaMYB121 with ALDH1 and DBR2 Promoters

**Figure 6:**
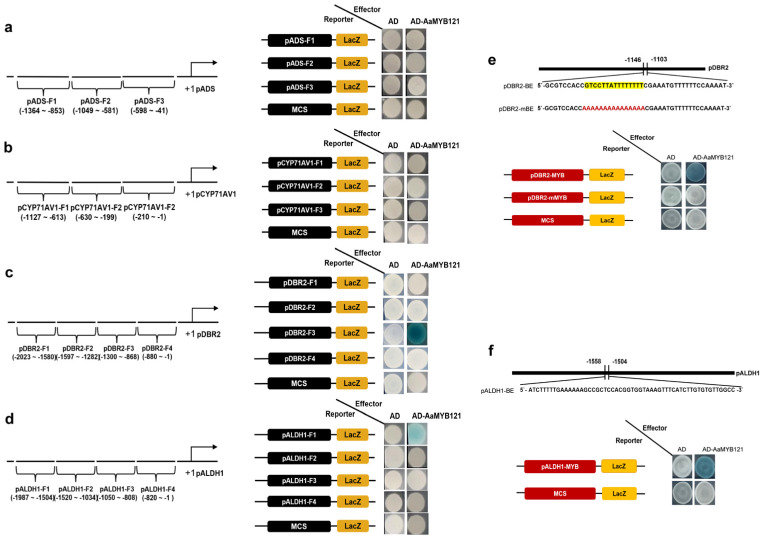
Direct interaction between AaMYB121 and the *cis*-elements in promoters. (**a**–**d**) Y1H analysis of AaMYB121 interaction with *ADS*, *CYP71AV1*, *DBR2*, and *ALDH1* promoters. pADS, pCYP71AV1, pDBR2, and pALDH1: the bait vectors harboring the promoters of the respective genes; F1~F4: different fragments of promotes; MCS, multiple cloning sites; AD: the empty prey vector (pB42AD); AD-AaMYB121: the prey vector with *AaMYB121*. The empty prey and bait vectors were included as controls. Potential *cis*-element positions (distance from the translation initiation site, ATG, which is set as +1) were indicated. (**e**) Specific binding of AaMYB121 to *DBR2* promoter. pDBR2-BE: the binding element (BE) in *DBR2* promoter; pDBR2-mBE: the corresponding mutated element. (**f**) Specific binding of AaMYB121 to *ALDH1* promoter. pALDH1-BE: the BE in *ALDH1* promoter.

To investigate the direct involvement of AaMYB121 in orchestrating artemisinin biosynthesis, we undertook yeast one-hybrid (Y1H) assays [32]. Due to the discrepancies observed between the Dual-LUC assay and the LUC chemiluminescence data, it was necessary to re-evaluate the promoter activities of critical genes (*ADS*, *CYP71AV1*, *DBR2*, and *ALDH1*) in artemisinin biosynthesis by adopting this method in yeast cells. To achieve this, we meticulously fragmented the promoter regions of these genes and incorporated each fragment into the pLacZ vector, constructing bait vectors for Y1H assays (Figure 6a–d). Meanwhile, the coding *AaMYB121* sequence was recombined into the pB42AD vector harboring the GAL4 activation domain to acquire the prey vector (Figure 6a–d). After constructing the bait and prey vectors, we co-transformed both into yeast cells, and evaluated their interaction by monitoring the LacZ enzymic activity, a reliable indicator of protein-DNA binding [33]. Negative control was conducted with empty bait and prey vectors. Our results revealed that AaMYB121 did not activate *LacZ* expression in yeast cells with the promoter fragments of *ADS* or *CYP71AV1* (Figure 6a,b), indicating that AaMYB121 does not directly bind to these promoter regions. However, AaMYB121 was able to activate *LacZ* expression in yeast cells with the F3 fragment (−1300~−868) of the *DBR2* promoter and the F1 fragment (−1987~−1504) of the *ALDH1* promoter (Figure 6c,d). The findings provided evidence that AaMYB121 directly binds the promotes of *DBR2* and *ALDH1.*

To refine our understanding of the binding specificity, we further subdivided the promoter fragments into smaller regions and performed additional Y1H assays. Through rigorous experimental procedures and data analysis, we discovered that AaMYB121 specifically binds to conserved elements within the *DBR2* promoter (−1146~−1103) and *ALDH1* promoter (−1558~−1504) (Figure 6e,f). More importantly, when mutations were introduced into this 15 bp AT-rich element (5′-GTCCTTATTTTTTTT-3′) within *DBR2* promoter, transcriptional activation was completely abolished (Figure 6e), indicating the mutated element (5′-AAAAAAAAAAAAAAA-3′) failed to support AaMYB121 binding, whereas the intact element was essential for AaMYB121 binding. The findings underscored the specificity of AaMYB121 binding to this particular *cis*-element, further confirming its critical role in regulating these genes.

**Figure 7:**
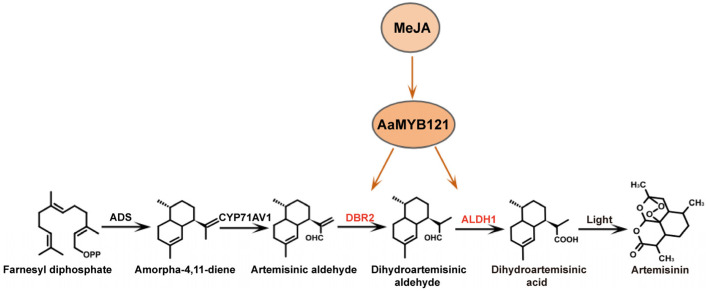
Regulatory mechanism of AaMYB121 in artemisinin biosynthesis. In response to the MeJA signal, AaMYB121 regulates the biosynthesis of artemisinin by specifically binding to the promoters of *DBR2* and *ALDH1*.

In conclusion, our findings provided compelling evidence for the direct involvement of AaMYB121 in regulating artemisinin biosynthesis, and its interaction with the *cis*-elements of *DBR2* and *ALDH1* promoters. Additionally, we found that AaMYB121 is responsive to the MeJA signaling pathway and that overexpressing *AaMYB121* significantly enhances the generation of artemisinin and dihydroartemisinic acid. Therefore, we proposed that in response to the MeJA signaling pathway, AaMYB121 promotes artemisinin biosynthesis through specific binding to the promoters of *DBR2* and *ALDH1* (Figure 7). These findings offered important insights into the transcriptional regulation of artemisinin biosynthesis and suggested exciting possibilities for further exploration of the molecular mechanisms underlying this vital pathway.

## 3. Discussion

MYB TFs constitute a vast protein family, characterized by one to four MYB repeats, each approximately 50 amino acids in length [10,11,12]. Depending on the quantity and sequence similarity of these repeats, MYB TFs are grouped into various subfamilies, namely R1-MYB, R2R3-MYB, R1R2R3-MYB, and 4R-MYB proteins. Among them, the R2R3-MYB subfamily holds a crucial position in regulating secondary metabolism, including artemisinin biosynthesis [12]. In *A. annua*, R2R3-AaMYB1 TFs have been found to regulate artemisinin production by promoting GSTs initiation, development, and upregulation of key artemisinin biosynthetic genes, such as *ADS* and *CYP71AV1* [14,19]. In this study, we identified a novel R2-MYB-like TF, AaMYB121, through clustering analysis of MYB family genes and artemisinin pathway markers. AaMYB121, which responds to MeJA signaling, significantly enhances artemisinin generation by directly activating the expression of two critical genes, *DBR2* and *ALDH1*, involved in the downstream stages of artemisinin biosynthesis. These findings not only broaden the scope of potential targets for enhancing artemisinin synthesis but also uncover new regulatory layers within the pathway’s transcriptional network.

The functional uniqueness of AaMYB121 is intricately associated with its structural features. Unlike typical MYBs, AaMYB121 possesses an extended C-terminal region comprising 496 amino acids, which likely enables it to interact with a wider array of regulatory proteins and target specific *cis*-elements. Notably, AaMYB121 preferentially binds to a longer AT-rich element (5′-GTCCTTATTTTTTTT-3′) in the *DBR2* promoter, a sequence distinct from the (5′-AACNG-3′)/(5′-ACCTAC-3′) element recognized by other R2R3-MYBs [34,35]. This unique binding preference implies a specialized regulatory ability. In addition, the stronger activation of AaMYB121 to the *DBR2* promoter could indicate a stronger specificity for this specific *cis*-element. The extended C-terminal region may also facilitate interactions with co-regulatory proteins, contributing to the indirect regulation of upstream genes like *ADS* and *CYP71AV1*. In *AaMYB121*-overexpressing lines, we observed an upregulation of these genes, indicating that AaMYB121 could modulate their expression via indirect pathways.

While indirect regulation might be linked to co-regulatory pathways, it is also plausible that hormone signaling plays a role in regulating *ADS* and *CYP71AV1.* The responsiveness of AaMYB121 to MeJA and the established synergy between JA and MYB TFs in secondary metabolism support this possibility [15]. For example, in *A. annua*, AaMYB108 is regulated by both light and JA, interacting with AaJAZ8 and AaCOP1 in the JA and light signaling pathways [15]. These observations suggest that AaMYB121 may be part of a broader regulatory network, involving multiple signaling pathways that coordinate artemisinin biosynthesis and other aspects of secondary metabolism. To further elucidate the regulatory network of AaMYB121, future studies should focus on identifying its interaction partners using techniques like yeast two-hybrid (Y2H) screening and other proteomic approaches [34,36]. These analyses could uncover additional layers of regulatory complexity, including potential interactions with other transcription factors and co-regulators that contribute to the precise control of artemisinin biosynthesis. Such studies will provide deeper insight into how AaMYB121 coordinates multiple signaling pathways to control secondary metabolism, providing a clearer picture of the molecular mechanisms underlying artemisinin biosynthesis. This will ultimately inform strategies to enhance artemisinin production, benefiting both plant metabolic engineering and pharmaceutical applications.

## 4. Materials and Methods

### 4.1. Plant Materials and Growth Conditions

Wild-type (WT) *Artemisia annua* and *Nicotiana benthamiana* seeds were sourced from Southwest University (Chongqing, China) [18,27,28]. The sterilization of *A. annua* seeds was achieved by first immersing them in 75% ethanol (1 min) followed by a 25% NaClO treatment lasting 10 min. After rinsing thoroughly with sterile water, the seeds were sown on a MS solid medium and germinated in a growth chamber at 25 ± 1 °C under a 16 h light/8 h dark cycle. For *N. benthamiana*, the seeds were directly sown in a soil blend composed of perlite, vermiculite, and needle moss (1:6:3) to ensure proper drainage and aeration. These plants were cultivated under the same temperature and light conditions. After four weeks, *A. annua* was prepared for genetic transformation, while *N. benthamiana* was selected for Dual-Luciferase (Dual-LUC) and subcellular localization analysis.

### 4.2. Co-Expression Analysis of MYB TFs and Artemisinin Biosynthetic Genes in A. annua

Co-expression and clustering analysis were executed following previous research [16,18]. Gene accessions of *AaMYB TFs* were extracted from the *A. annua* proteome data (NCBI ID: txid35608) using HMMER-3.0 (http://www.hmmer.org/, accessed on 8 February 2025) and Pfam-derived MYB domain profiles (http://pfam.xfam.org/, accessed on 8 February 2025) database. To identify potential AaMYBs associated with artemisinin biosynthesis, raw RNA-seq data for various tissues were obtained from the NCBI Sequence Read Archive (SRA) database (epidermis: SRR6472945; bud: SRR6472946; seed: SRR6472947; flower: SRR6472948; young leaf: SRR6472941; old leaf: SRR6472942; stem: SRR6472943; root: SRR6472944) [18]. Transcript abundances were quantified with SALMON [37]. Hierarchical clustering of *AaMYBs* and artemisinin biosynthetic genes was performed using the R package PHEATMAP v.1.0.10 (https://rdrr.io/cran/pheatmap/, accessed on 8 February 2025).

### 4.3. Treatment of MeJA and ABA

According to the prior findings, thirty-day-old WT *A. annua* received foliar applications of either 300 μM MeJA (methyl jasmonate) or 10 μM ABA (abscisic acid) [18]. After that, temporal leaf sampling occurred at various time intervals for gene expression analysis.

### 4.4. Development of Transgenic A. annua Plants

To engineer the pHB-AaMYB121 construct, the coding sequence of *AaMYB121* was directionally ligated into the pHB backbone plasmid using the constitutive 35S promoter. The recombinant plasmid was introduced into *A. tumefaciens* EHA105 and then employed to infect *A. annua* using established protocols [18,26]. Subsequently, explants were transferred to a hygromycin-supplemented selection medium for transgenic plant screening. Cultures were maintained under selection until shoot regeneration, after which regenerated shoots were transferred to a rooting medium to promote root development. The plantlets were acclimatized in pots containing a mixture of vermiculite and peat for continued growth. Molecular validation of transgenic lines involved PCR amplification with gene-specific primers (Table S1), with comprehensive methodological details provided in prior work [27].

### 4.5. RNA Extraction and Quantitative Real-Time PCR (qRT-PCR)

Tissue-specific sampling encompassed root, stem, old leaf, young leaf, bud, and flower from WT *A. annua* plants for *AaMYB121* expression profiling. Phytohormone-treated leaves and transgenic/WT comparative samples were also gathered to evaluate the expression of *AaMYB121* and artemisinin biosynthetic genes. Total RNA isolation employed the plant RNA Extract Kit (Tiangen, Beijing, China), followed by DNase-treated cDNA synthesis using FastKing RT reagents (Tiangen). qRT-PCR detection was performed using PowerTrack SYBR Green Master Mix (Thermo, Waltham, MA, USA), with the following thermal cycling conditions: step 1, 95 °C for 10 min; step 2, 95 °C for 15 s; step 3, 60 °C 30 s; step 2 to step 3, 40 cycles. Then, the relative expression of genes was determined through qRT-PCR, with *β-actin* as the reference gene. Expression fold changes were analyzed via the 2^−ΔΔCT^ algorithm [38]. All qRT-PCR analyses were performed with three biological replicates. Relative primers were listed in Table S1.

### 4.6. Measurement of Artemisinin and Dihydroartemisinic Acid

Leaves from transgenic and WT *A. annua* plants were processed for artemisinin and dihydroartemisinic acid (DHAA) extraction. Chromatographic analysis was performed via High-Performance Liquid Chromatography (HPLC) following published methodologies [27,39]. The standards for artemisinin and DHAA were acquired from Sigma-Aldrich (St. Louis, MO, USA). Triplicate biological replicates per genotype ensured experimental reproducibility and data robustness.

### 4.7. Subcellular Localization of AaMYB121

The coding sequence of *AaMYB121* was inserted into the pHB-GFP construct and introduced into *A. tumefaciens* GV3101. Transformed bacterial cells were infiltrated into *N. benthamiana* to investigate protein localization of AaMYB121. An empty pHB-GFP vector served as the control. After 48 h of incubation in the dark, the leaves were injected with 1 μg/mL DAPI (4′,6-diamidino-2-phenylindole) for nuclear staining. Cellular distribution patterns were examined with a Leica TCS SP5 laser confocal microscope (Leica Microsystems, Wetzlar, Germany). GFP signals from the AaMYB121-GFP fusion protein along with DAPI-stained nuclear were visualized simultaneously to confirm subcellular positioning. The primers are shown in Table S1.

### 4.8. Dual-Luciferase and LUC Luminescence Assay

Dual-LUC assays were conducted as described before [16,18,23]. Promoter regions of target genes were amplified from the genomic DNA of *A. annua* and incorporated into the pGreenII0800-LUC vector to construct reporter plasmids (primers in Table S1). The recombinant pHB-AaMYB121-GFP, which fuses *AaMYB121* to the N-terminal of *GFP*, was used as the effector. The pHB-GFP vector was utilized as the negative control. The transformation of *N. benthamiana* was carried out using the *Agrobacterium*-mediated infiltration method [25]. After infiltration, leaves were kept in darkness for 48 h prior to luminescence measurement. Relative LUC activity was quantified with a GloMax 20/20 Luminometer and Dual-Luciferase Reporter Assay System Kit (Promega, Madison, WI, USA), following standardized protocols. Five independent biological replicates were performed for each experiment. The promoter regions are indicated in Table S2.

For LUC luminescence imaging, *N. benthamiana* leaves were injected with 1.0 mM _D_-luciferin potassium salt (Beyotime Biotechnology, Beijing, China), and incubated in darkness for 10 min to permit substrate conversion. Subsequently, the leaves were collected and placed in an automated chemiluminescence imaging system (Tanon-5200, Shanghai, China) to capture the luminescence signal for living plants.

### 4.9. Yeast One-Hybrid Assay

The procedures for the Y1H assay were adapted from previous protocols [32]. First, truncated promoter fragments of *ADS*, *CYP71AV1*, *DBR2*, and *ALDH1* were amplified from the genomic DNA of *A. annua*. These DNA fragments were then inserted into the pLacZ vector as bait elements. The prey construct was created by fusing the coding sequence of *AaMYB121* with the GAL4 activation domain in the pB42AD vector. The negative control groups included empty pB42AD and pLacZ vectors. Yeast strain EGY48A was co-transformed with bait-prey plasmid pairs. Transformants were first selected on SD/-Trp/-Ura agar, then subjected to interaction verification using X-gal-containing indicator media, where positive interactions triggered β-galactosidase-dependent chromogenic reactions. Initial Y1H data guided the iterative design of truncated promoter derivatives for precise binding motif characterization of AaMYB121. Primers were indicated in Table S3.

## 5. Conclusions

AaMYB121, a newly identified MYB-like member in *A. annua*, was found to directly regulate the expression of key artemisinin biosynthetic genes *DBR2* and *ALDH1*. Its responsiveness to MeJA and nuclear localization confirmed its role as a transcription factor. Overexpressing *AaMYB121* significantly enhanced the expression of *DBR2* and *ALDH1*, promoting the production of DHAA and artemisinin. Unlike canonical R2R3-MYBs, AaMYB121 features an elongated DNA-binding domain and targets AT-rich *cis*-elements in the promoters of these genes to activate their expression. The findings expanded the regulatory framework for artemisinin production and provided a strategic target for metabolic engineering aimed at improving artemisinin yields.

## Data Availability

The original contributions presented in the study are included in the article and Supplementary Materials, and further inquiries can be directed to the corresponding authors.

## Supplementary Materials

The following supporting information can be downloaded at: https://www.mdpi.com/article/10.3390/ijms26062441/s1.
