# Supplementary material for: AaMYB121, a Novel R2-MYB-like Transcription Factor, Regulates Artemisinin Biosynthesis in Artemisia annua
Source: Int J Mol Sci. 2025 Mar 9;26(6):2441. doi: 10.3390/ijms26062441 (PMC11942501; doi:10.3390/ijms26062441)
Supplement: Supplementary file 1 [file ijms-26-02441-s001.zip › ijms-3495618-supplementary.pdf]

Supplemental Information

Table S1. Primers for gene cloning, molecular detection and qRT-PCR.

Name	Sequence (5′ to 3′)
AaMYB121-PHB F	TGGATCCTCGAGCTGCAGATGTTTCTAGCCTCAAAAACAAC
AaMYB121-PHB R	GAGGATCAATTCGAGCTCTCATAGAGTGAACTCAAGATTTAG
AaMYB121-F	ATGTTTCTAGCCTCAAAAACAAC
AaMYB121-R	TCATAGAGTGAACTCAAGATTTAGC
Hygr-F	GCCTGAACTCACCGCGACGTC
Hygr-R	TTTCTTTGCCCTCGGACGAGTGCT
AaMYB121-PHB-GFP-F	GACGAGCTGTACAAGCTGCTGCAGATGTTTCTAGCCT
AaMYB121-PHB-GFP-R	CTAGAGGATCATTCGAGCTCTCATAGAGTGAACTCAAG
qAaMYB121-F	GCCATGGAGTCCAGCTTGAT
qAaMYB121-R	ACCAAGGAGTTGAACGGCAT
qADS-F	GGGAGATCAGTTTCTCATCTATGAA
qADS-R	CTTTTAGTAGTTGCCGCACTTCTT
β-actin-F	CCAGGCTGTTCAGTCTCTGTAT
β-actin-R	CGCTCGGTAAGGATCTTCATCA
qCYP71AV1-F	ACTGACCACTTCCATTGCTCTTG
qCYP71AV1-R	ACTTTCTGGCTAAATCCCTAACCC
qALDH1-F	CGGAGTAGTTGGTCACATCATTC
qALDH1-R	TTAATCACGCCATCAGGAACAC
qDBR2-F	ATCATCAACAAGCAAGCCCATTTC
qDBR2-R	GCGATAGTCTTCAACCACCTCTAG

Table S2. The promoters of artemisinin biosynthetic genes.

Gene	Sequence (5′ to 3′)
	TATGGTGTTTCAACGCTTATGAGTTGGTACATGTTATAACAATCCACAAATGTGCTTA
	AACAACCATTATAGCGTGACATGAATTAAAGGAAATGAGAAGAAAACAAATAGAA
	TCGTTACACCTTTATTTTTGTGTCGAATGAAAATGAAATCGTAAGCTCTATAGGAATT
	CCGCACTACACACATGGCTAAAACCGAATTCAAAATGCATCCTAGCCTTCTTGATTG
	ATCACCACATCGAATGGGAGGAAGAAAAGCAGCTTAGCCTCATTTTGGTGGAGATA
	AAAGTTCATACGCATTTTTATTTTGATGTTAGGGTTTTGAGAACAAAACCGGTTATGT
	ATTATTTTGATGGACTTGGGCTTGGGAAAAATTAAATGGGTCAATTGGGGAGTGTTTA
	GAAAGGCCCAAATGGATTTTAGTTGGGTTAGTTTAGTCCGTGTAGCTTGTGTCGAATA
	ATCGTTGCCCAAAAACAATTATCGGGTTTCGTTTAAATAATTCTCGTAGCCAAATTTA
	ATTTACAAAAATTAATTAGGGCTTATAATGACATGTTTGGTTCAAAATTTTAATTATA
	TCACATATTATACATATTACGGGTCGTAGTGAAATCGTTTAACGGTTTACGAAATTCG
	GGTCATTACATACAAGCACACCCTTAAATGTTACCATTTGATGGTTTATATAGTTATA
	CTAGGCGCCACATATGGGCCTTGATGGGCTTGGTGGTGTTAGACTTGCATTGGTTTAC
	ACTTTTATTATAAGCTTACACTATCCTAACACATTATCTATCATCATACAATGTCCAT
	AGCCCGATACTACTTTAATACAATTACTATCACACGTTAGAAGGTTCTTAATACAATA
	ATGACTATCACTTCTTTTAATGCTGACACATATTGTGCTTGAGTCATATTTGAAGACA
	ATAATATAGATTACATTTGATCTTAAATTGTAGTTAGAAATAACAAGAAAACATGGA
	TTGGTTGTTTTATAAGATATAGGACAACATGTAGATTTAATAAATATTGTGAGTAAGA
	CTAATATGTTTTTGAAATGTTGACCCATTCATCACGCAGGCCGTGTTGGCTAGTGTTA
	AAGTAATACATTGTTTAATAAAATTCAATTGTTTACCGGTTGTATGAAAATATTTTAC
	CCCATAAAAAAAATCACTTGTTTAGGATTTAGGTCTTATTGAGTGTGCTACTGTCAAA
	ATGATTTACCCCATAAAAAAATTCAATTGTTTAGGATTTAGGTCTTATTGAGTGTGCT
	ACTGTCTTTCTATAAAATCTAGTAACTCTCCATCAAGCTAAGAGCAACTCTAGTAAAT
ADS promoter	TAAACCACATATATTCAAAGTTTTGAAAATCGGATCC
	TTTTCTGACCTGCCAACCTGACAACCCGAACCCGCCAATCCGAACCCGACCCGAATT
	GCCACCCCTAAATACATGTAAGCATTTGGATTGCAATAAACACTACAAAACTCATAG
	ATCCGTATATTTACAAAACTAAGAAAAAAGACAAAGCTAATCTAAACAATACAAAA
	CTCTAAAAGCCGATTGACACAACTACAAAACATGACGCACAAGCAATACAAAAATT
	AGGGGTGTTCAAGCAACACTACAAAACATCATCACTACTGACATCTTTATGTGTTTTA
CYP71AV1	ACCCACTCATAAGTATTATACTAATACATGTTACAAGCCTCTCATGTATTATACAACT
promoter	GACATCTTGTTTTTATCATCATTTATGTAATGGCCCTGTGACAATAAACAAAATGATA

	GCCATATAATCCACTTGCGTCGTTGGCTTTTCAAGTTGATACCATCTAACATTCTAAC<br>ACACGTATAGCAAACATAATGAAGTAAAATACACAAAATCAAATTAGTATATTGGT<br>CAAATAAGGATAGCAATGGTCCATTTTTGACAAACTCAGTAGCATAACATGTTTTAT<br>ATCTAATAGCTTTTTCCAGTTTATAAATTCATATCTTTTACGTGTCAATTTCTTGACTTT<br>AACACTTATATAAGTTATAATCTTCATTTTTTGGCAACAACTATTATATTTTTAGGTCA<br>TGTTTAATTTTAATTTTGACATTAAGTTTACATAACTTTAATACTATAATACACTACTT<br>TCAGTTTGGATGAACACCTTGATTTCCATTATAACATCTCTGACGTCAACTTTTGAATC<br>TAATTAAGGTCACTCACAAACTAAAACTTTCGGCATCCCCTTATTTTGTTGAATGTAA<br>CCAAACCACGTAAGTTTTCCTTTCTTGGTCAATGCATAAATGTAAAAGATGGCATGTT<br>ATATTAACTAAACAATTAATACGAAATAATTTTTCATAATAAAACTGGACATAAATG<br>TCAAGAGTGGACTACTGCACATTCAATGAGGGTGTCATTGAAATTCCAAATGGGGTA<br>GCACATATTCAATTCCGCATCAACCCCATACAACAATATAAACAACCTTAATTAACA<br>TTTGACACCATGAAGAGTATACTAAAAGCATG
DBR2 promoter	CCCCAATGGGTTGGTCTAAGGACCACTGAAAGCTTGTGATATTTTTTTCAAATGACCC<br>AGATAGTTGGATCGAAAGATGTGATTTACATATTAGAAAAACTAAAGAACTTCAAG<br>ATAGAAAAGTTCACGACGACCTTCGACAAGACCTTGTGGAGCGTTTATGGAACTTAA<br>ATCATTAGTTTGTATTTTTTTTAAATGTAATGTGTGTTTTTTTAATTAATGAAGTTTATT<br>TTTAGTTAATATTGTGTATGTTTTATTTTATGTGAAAAAAAAAAAAAAAAAAAAAAA<br>AAAAAAAAAAAAAAAAAATGGCCGACGGCACCTGAATTTCTGGTGCCAACTGTAAC<br>AACTAACTACGTCATCAAAAAAAATAATTAAAGCACGAAAATAAAAATAATAATTTA<br>TTAAAAGATTAGAAAAAATCTCGCGCGTGCGCTGGCATTATATCTAAGTATAGTAGA<br>AAGCAAATACGTAAGCCCTTTAATTTATTTCCAATAATGGCATTTCGTTTATCAATTTT<br>GTGTGATTGGTGGATATTACTTTTAACCTATAAATTAATATTTATCATCAACTGATCAT<br>GATAATAATGGCAGAAACAACGATAGAAAAACCAACCTCCCTACATCAAAATCACT<br>TGTTGCGACGGCATAAAGTGAGTCTGGCTTAACATTTACAGTTGTGTATATTAATAAA<br>GTTTGAAGTTTCCTTTTAGGGCAAATAACCTGTTAAAGCACTATACTTGTCATAAAGT<br>GAGTTTCGCGTGCTGAACAAAAAAAAATCATTGTTTGTACACTAATGTTGACACTTTTG<br>TCTTTTTTTAAGTATTATTACCATTTTACCTTTCCAAATTACATAAATGGTCCCTGTCGT<br>TTGGAGAAAATTGCGTCCACCGTCCTTATTTTTTTTCGAAATGTTTTTTCCAAAATTTTT<br>TTTTTCTAAATTCTTTTATTTATGATTTTTTTTAAATTCTTTTTTTTTTTTTGAATTTTTTTT<br>TGTTTGAAATAATTTTGGTTAGAAATTTTTTTCGTGACATGTGAAATTTTTTTTGACCG<br>CTTTCTGGTCCGAATCATCAAAAGTGTAAAACACAAAAGTTGTCCGAAATAATTTTT<br>ACATCAAGACTCGTGTGATTTTAACCTGAAAATGCAAAACAAGGGCTGTTTATTACA<br>AAAACTTAAGTGGTTTTCGACCAAATTCATAATTATAAACTCTTGTAAATTTTATAAC

	TTGTGTAAATAATCGAACTTATTTGAAAAAAAATAGGGACAAAACATAAGAAAAAA
	AATAATTTCAAAAAAATTCACTTAAAAAAACATAAAAAAAATTCACTTAAAAAAAA
	ATATTTTTTTTCAAAAAAAAAAAATTCACTTAAAAAACATAAAAAATTTCACTAAAAA
	AAATTCCCATAAAAAAAAATTCACTTAAAGATTTTCTAAAAAAAATATTTCAGAAAAT
	TTTTTTGAAAAAAATAAGAATTTCAAAAAAAAAAAAATGGAAAAAAAAGAGACGG
	TGGACGCAATTTTCTCTAAACGACAGGGATCATTTTGACCCGTCAAGTCAACATGGT
	GCCACTGATACATCTACAAAGAGTTGAAAAAACTATATATCTACATAATAATTTGTC
	AATTAAACGTGAAAAGTAAAGAATAAAAATAAAAGATTATGATACAATATGATTGA
	ACCCTTTGACAAGTTATTGTGTGAATATAATTTCGTAAATCTTTTGTGATACGTGATAT
	TAGAAAATTGAGCATATATTTGATTTTCAATAACTAGTTTTTTTTTTTTTTTTTTTTTT
	TTTTTTTTTTTTTTAAATGAATGTTACTTTATGACATGTAAGCCGAAGCAAGTCAAAA
	CAAGTATGTTTATTTTATGTTCGAGAACACGGTGTCTAAAATGGTGATAGTTTGACTT
	TGAAACAACGCTGTATCACTCTTTGTGGTACTATTTAATATTGTCTTCAACACATCAC
	ACTTTACCAACTCCATCACTGATCTTGATCCTGGTCAACATCAAACTCAATACCATGG
	AAGGGAAGGAGTATTATACTATTAGATTGAGATTCTCCCAAGTTAGAATGGATATAT
	ATTCCCTCACATACACAACTGTGGGGTCTACTGCGGGACCCAATATTTCTTTTGTTTGT
	GAAGTAACATAACCTATCAAGTTCATTCATACTTGAGAGAACCTCAATCTTATATTTT
	TACTTTGAGCATTGCTAAAGCCTTCTATAATTTCCTCCTATACACACAACAATATAAC
	TTTTTTCACTTATTCTCTCTCTGCATCTAAGCCGTTAATTCTGATCAGAATCCTGTGAG
	TAAGAGGATAAATAGAGACAATTTGTAAAAGGAAATAACATTTTCCTTTTACTTTTG
	GTTGATTGCATTATCTGGCCCCACCGTAAACAATCAAATGCCTTTTGTTTTCATATTTT
	GCGTGTTTAGATTTTCCTCGTCATCTTTTTGAAAAAAGCCGCTCCACGGTGGTAAAGT
	TTCATCTTGTGTGTTGGCCGAACTTACAATAGTGACATTGAACCCTCGAATACGTTCG
	AAGATCTCGAAATGATCTTCCAGTTCCGGGGAGAATTCGCAAAACTCCATTTCCATC
	AAGAATTGAATGGACTTTTCTCGTATTTCGTCCAGAAAATCTTAAGTTGCGCACTTAC
	ACAATTTGTAAGCATCTTTTCATTCTTATTCAGGCTATATATTAGTATGTTTTTAACAA
	TTATTTTTCCCGACTCTCTATCGATTTTTTTTCTTCCTTTTTTTTCTAGAAATGTATCATC
	ATTCGTATAAAAGAGAAAAAGTCAATTCGGAGGGGCTAAGGCCAACCATAAAACAT
	AGCCGCCAGCCCAATACTCCAAAAGAGCACGCCAAGTATGTATTCTAGATTTGTAGC
	CATTACTACTGGAACCGATATTTGCGAAAACCAAACTAAGCAGAACTGAATTCGATT
	AATGTACGTTTTTGGTTCTTTCATGTAACCAAACTGAACTAAAGACCCTAATATATGT
	TTGTATGCAATAATCCTTAACATGTTTACCATAAACAAAGTGAAACATCCGTAAACT
	TACATGCACAATTGGAGCTCGATTTTGTTTCTGTTTATGATGACAAAGTTTTTCAAGTG
ALDH1 promoter	ACAAAACTAACACAAGAAAACTAGAAAAAACAGAAAACGTAGCCGATACCTTCTC

	TCACTCTCTCCCTTTGTGCTTTTATGATTTTTCTCCCTATTTTACTATAAAATGTTATGT
	GAACACACTAACATAATACCAATTAGATTACCCAAGGCCTTTGGTCTATTGGTATGT
	GAGTTGCCCACCAACCTAGAGGTTGTGGGTTCAAGCCTTACTGGAGGTATAGGATGT
	CTAAAAAATTGCCGTTAAAAAAAAAAACAGAAAAAAGAACAATTATATCCTTTTTA
	TTTTTTTATTATTTATTTATTTTTATTTTTTTGGTAAAAAAGTTAACCCAGTAAAGTATT
	GTTACAATTATATCCCTTTTGGTAACTAAATATTACAATAAAGGCCTATAAATCTTTT
	ATATAATAAAACAAAACCCCTAGTGGGTACTTTCGTATTTCGAAAAAAAGAATATTT
	CCTTATTAGGTAATATTTTAATTATTATTTTTTAATTTAAAATTTTAAAAATTTGTTTTC
	TTGATATACTACACAAGTAGAAGATAAATATAAAAAATATAGATAAGATGTAACTA
	ACAATAGGATTAAATTTTTTTAATGATTATCTTTAATAATAAACAAATATAATTAAAA
	ATCATAAATCAAACAAAATAAAGATTAAATTATTTGTTTTTAAAAAAATAAAAATGT
	ATAACTCAAAAAAAATTAAAAAAAATCCGCGCGTGCGCGGGCATTATAACTAGTAG
	TCCTATGACATTCGACCCTTGAATAAAAATTTCCCTTTTGATAACTAAATATTATAAT
	AAAGGCCTATAAATAGTCCTATGCAAAGCCTTAAGTCATCATTCGACCCTTGAATAA
	AAATTTCATAAAAAAACAAAGATG

*The red marked region represents the specific binding motif of AaMYB121.

Table S3. Primers for Y1H assays.

Name	Sequence (5′ to 3′)
MYB121-PB42AD-F	GATTATGCCTCTCCCGAATTCATGTTTCTAGCCTCA
MYB121-PB42AD-R	GAAGTCCAAAGCTTCTCGAGTCATAGAGTGAACTCAAG
pADS-F1-placZ-F	GAATTCGAGCTCGGTACCTATGGTGTTTCAACGCTTATGA
pADS-F1-placZ-R	CAGAGCACATGCCTCGAGTTTGGCTACGAGAATTATTTAAACGA
pADS-F2-placZ-F	GAATTCGAGCTCGGTACCGGGTTTTGAGAACAAAACC
pADS-F2-placZ-R	CAGAGCACATGCCTCGAGTAATGTGTTAGGATAGTGTAAGCT
pADS-F3-placZ-F	GAATTCGAGCTCGGTACCCACTATCCTAACACATTATCTATCA
pADS-F3-placZ-R	CAGAGCACATGCCTCGAGACTAGAGTTGCTCTTAGC
pCYP71AV1-F1-PlacZ-F	GAATTCGAGCTCGGTACCTTTTCTGACCTGCCAACCTGAC
pCYP71AV1-F1-PlacZ-R	CAGAGCACATGCCTCGAGGACCAATATACTAATTTGAT
pCYP71AV1-F2-PlacZ-F	GAATTCGAGCTCGGTACCCAAATTAGTATATTGGTCAAATAAGGA
pCYP71AV1-F2-PlacZ-R	CAGAGCACATGCCTCGAGAATATAACATGCCATCTTTTACA
pCYP71AV1-F3-PlacZ-F	GAATTCGAGCTCGGTACCAAATAAGGATAGCAATGG
pCYP71AV1-F3-PlacZ-R	CAGAGCACATGCCTCGAGAATATAACATGCCATCT
pALDH1-F1-placZ-F	TGAATTCGAGCTCGGTACCAAGGGAAGGAGTATTATACT

pALDH1-F1-placZ-R	CAGAGCACATGCCTCGAGGGCCAACACACAAGATGAAACT
pALDH1-F2-placZ-F	TGAATTCGAGCTCGGTACCCATCTTGTGTGTTGGCCGAAC
pALDH1-F2-placZ-R	CAGAGCACATGCCTCGAGACATGAAAGAACCAAAAACGTAC
pALDH1-F3-placZ-F	TGAATTCGAGCTCGGTACCGAACTTACAATAGTGACA
pALDH1-F3-placZ-R	CAGAGCACATGCCTCGAGACATGAAAGAACCAAAAAC
pALDH1-F4-placZ-F	TGAATTCGAGCTCGGTACCATGATTTTTCTCCCTAT
pALDH1-F4-placZ-R	CAGAGCACATGCCTCGAGCATCTTTGTTTTTTATGA
pALDH1-F5-placZ-F	TGAATTCGAGCTCGGTACCAAGGGAAGGAGTATTATACTA
pALDH1-F5-placZ-R	CAGAGCACATGCCTCGAGGAGAGAGAATAAGTGAAAAAAGTTA
pALDH1-F6-placZ-F	TGAATTCGAGCTCGGTACCGAATCCTGTGAGTAAGAGGATA
pALDH1-F6-placZ-R	CAGAGCACATGCCTCGAGGGCCAACACACAAGATGAAACT
pALDH1-F7-placZ-F	TGAATTCGAGCTCGGTACCGAATCCTGTGAGTAAGAGGA
pALDH1-F7-placZ-R	CAGAGCACATGCCTCGAGAAGGCATTTGATTGTTTACGGT
pALDH1-F8-placZ-F	TGAATTCGAGCTCGGTACCCAAATGCCTTTTGTTTTCATA
pALDH1-F8-placZ-R	CAGAGCACATGCCTCGAGGGCCAACACACAAGATGAAAC
pDBR2-F1-placZ-F	GATGAATTGAAAAGCTTCCCCAATGGGTTGGTCTAAGG
pDBR2-F1-placZ-R	CATGCCTCGAGGTCGACGATATAATGCCAGCGCACG
pDBR2-F2-placZ-F	GATGAATTGAAAAGCTTTAAGTATAGTAGAAAGCAAATACG
pDBR2-F2-placZ-R	CATGCCTCGAGGTCGACATGACAAGTATAGTGCTTTAACAG
pDBR2-F3-placZ-F	GATGAATTGAAAAGCTTAAAGTGAGTTTCGCGTGCTGAAC
pDBR2-F3-placZ-R	CATGCCTCGAGGTCGACTAAACAGCCCTTGTTTTGCAT
pDBR2-F4-placZ-F	GATGAATTGAAAAGCTTTTACAAAAACTTAAGTGGTT
pDBR2-F4-placZ-R	CATGCCTCGAGGTCGACCCATGGTATTGAGTTTGA
pDBR2-F5-pLacZ-F	GATGAATTGAAAAGCTTCCCCAATGGGTTGGTCTA
pDBR2-F5-pLacZ-R	CATGCCTCGAGGTCGACCTAAAAATAAACTTCATTAATT
pDBR2-F6-pLacZ-F	GATGAATTGAAAAGCTTGTTTATTTTTAGTTAATATTGTG
pDBR2-F6-pLacZ-R	CATGCCTCGAGGTCGACGATATAATGCCAGCGCAC
pDBR2-F7-pLacZ-F	GATGAATTGAAAAGCTTGCGTCCACCGTCCTTATT
pDBR2-F7-pLacZ-R	CATGCCTCGAGGTCGACGTCACGAAAAAAATTTCTAAC
pDBR2-F8-pLacZ-F	GATGAATTGAAAAGCTTTTTTTTGTTTGAAATAATTTTGGTTAG
pDBR2-F8-pLacZ-R	CATGCCTCGAGGTCGACTAAACAGCCCTTGTTTTGC
pDBR2-F9-pLacZ-F	GATGAATTGAAAAGCTTGTTTATTTTTAGTTAATATTG
pDBR2-F9-pLacZ-R	CATGCCTCGAGGTCGACGAAATTCAGGTGCCGTCGGC
pDBR2-F10-pLacZ-F	GATGAATTGAAAAGCTTCTAACTACGTCATCAAAAAA

pDBR2-F10-pLacZ-R	CACATGCCTCGAGGTCGACGATATAATGCCAGCGCACG
pDBR2-F11-pLacZ-F	GATGAATTGAAAAGCTTGCGTCCACCGTCCTTATTTTTTTTC
pDBR2-F11-pLacZ-R	CATGCCTCGAGGTCGACAAAATCATAAATAAAAGAATTTAGAA
pDBR2-F12-pLacZ-F	AATGATGAATTGAAAAGCTTATGATTTTTTTTAAATTCTT
pDBR2-F12-inpLacZ-R	CATGCCTCGAGGTCGACGTCACGAAAAAAATTTCTAACC
pDBR2-F13-pLacZ-F	AAATGATGAATTGAAAAGCTTTTTTCGTGACATGTGAAATT
pDBR2-F13-pLacZ-R	CATGCCTCGAGGTCGACAATTATTTCGGACAACTTTTGTG
